# Complete Genome Sequence of Bacillus megaterium Myophage Moonbeam

**DOI:** 10.1128/genomeA.01428-14

**Published:** 2015-01-15

**Authors:** Joshua N. Cadungog, Brontee E. Khatemi, Adriana C. Hernandez, Gabriel F. Kuty Everett

**Affiliations:** Center for Phage Technology, Texas A&M University, College Station, Texas, USA

## Abstract

Moonbeam is a newly isolated myophage of Bacillus megaterium, a common Gram-positive bacterium that is routinely used for large-scale protein production. Bacteriophages have potential to be useful tools for industrial applications. Here, we describe the complete genome of Moonbeam and describe its features.

## GENOME ANNOUNCEMENT

Bacillus megaterium is a soil-dwelling bacterium that is commonly used for the production of recombinant proteins (1). Additionally, it is being investigated for use in biomineralization and bioremediation of soil contaminated with petrochemicals (2, 3). B. megaterium phages could provide a useful tool for manipulating these systems. Here, we describe a novel myophage, Moonbeam, which was isolated against the asporogenic B. megaterium strain Km sp.

Bacteriophage Moonbeam was isolated from a soil sample collected in College Station, TX. Phage DNA was sequenced in an Illumina MiSeq 250-bp paired-end run with a 550-bp insert library at the Genomic Sequencing and Analysis Facility at the University of Texas (Austin, TX, USA). Quality controlled, trimmed reads were assembled to a single contig at 32.6-fold coverage using Velvet version 1.2.10. Contigs were confirmed to be complete by PCR. Genes were predicted using GeneMarkS (4) and corrected using software tools available on the Center for Phage Technology (CPT) Galaxy instance (https://cpt.tamu.edu/galaxy-public/). Morphology was determined using transmission electron microscopy performed at the Texas A&M University Microscopy and Imaging Center.

Bacteriophage Moonbeam has a 161,239-bp double-stranded DNA genome, including 1,980-bp terminal repeats, as determined by processing of raw sequencing data using PAUSE (https://cpt.tamu.edu/pause/). The unit genome has a GC content of 40.2%, a coding density of 90.6%, and 3 tRNA genes. Of the 231 predicted genes, 79 are hypothetical novel, and 88 are hypothetical conserved. Comparison using EMBOSS Stretcher revealed 47.0% identity with paradigm phage SPO1 (NC_011421) (5).

Several functional proteins were identified using BLASTp and InterProScan analyses (6, 7). Genes encoding structural proteins include a capsid protein, portal, prohead protease, tail proteins, tail chaperones, tape measure protein, tail proteins, and multiple components of the baseplate. The tail chaperone had an unusual +1 frameshift to its secondary product, where most Caudovirales use a −1 frameshift to encode their secondary tail chaperone (8). Notably, the terminase is interrupted by two group I introns containing embedded homing endonuclease genes. Homing endonucleases are commonly embedded within group I introns, which catalyze their own splicing from an mRNA transcript (9, 10). An interruption of a terminase by a group I intron has also been reported in Lactobacillus phage LL-H (11). The terminase is homologous to the terminases of several Bacillus phages, including Bcp1, 1102phi1-3, and Spock (12–14). Genes encoding replication fork components, including primase, helicase, and DNA polymerase, were identified, as were several DNA-binding proteins. As in SPO1, the DNA polymerase gene of Moonbeam is interrupted by group I intron-embedded homing endonucleases, although SPO1 DNA polymerase contains one intron while Moonbeam encodes two (15). Genes for biosynthesis proteins were present, such as thymidylate synthase, dihydrofolate reductase, ribonucleotide reductase, and dUTPase. A class-II holin gene (two transmembrane domains in an N-in, C-in topology) was identified, although the antiholin was not found (16). Moonbeam encodes an FtsK/SpoIIIE DNA pump, although its role in the phage infection is unknown (17).

### Nucleotide sequence accession number.

The genome sequence of phage Moonbeam was contributed to GenBank with the accession number KM236246.
